# Comprehensive RNA-Seq profiling to evaluate lactating sheep mammary gland transcriptome

**DOI:** 10.1038/sdata.2016.51

**Published:** 2016-07-05

**Authors:** Aroa Suárez-Vega, Beatriz Gutiérrez-Gil, Christophe Klopp, Gwenola Tosser-Klopp, Juan-José Arranz

**Affiliations:** 1 Departamento de Producción Animal, Facultad de Veterinaria, Universidad de León, Campus de Vegazana s/n, León 24071, Spain; 2 INRA, Sigenae, UR875 Biométrie et Intelligence Artificielle, BP 52627, Castanet-Tolosan Cedex 31326, France; 3 INRA, UMR1388 GenPhySE (Génétique, Physiologie et Systèmes d’Elevage), Castanet-Tolosan F-31326, France; 4 Université de Toulouse, INP, ENSAT, GenPhySE (Génétique, Physiologie et Systèmes d’Elevage), Castanet-Tolosan F-31326, France; 5 Université de Toulouse, INP, ENVT, GenPhySE (Génétique, Physiologie et Systèmes d’Elevage), Toulouse F-31076, France

**Keywords:** RNA sequencing, Gene expression, Animal physiology

## Abstract

RNA-Seq enables the generation of extensive transcriptome information providing the capability to characterize transcripts (including alternative isoforms and polymorphism), to quantify expression and to identify differential regulation in a single experiment. Our aim in this study was to take advantage of using RNA-Seq high-throughput technology to provide a comprehensive transcriptome profiling of the sheep lactating mammary gland. Eight ewes of two dairy sheep breeds with differences in milk production traits were used in this experiment (four Churra and four Assaf ewes). Milk samples from these animals were collected on days 10, 50, 120 and 150 after lambing to cover the various physiological stages of the mammary gland across the complete lactation. RNA samples were extracted from milk somatic cells. The RNA-Seq dataset was generated using an Illumina HiSeq 2000 sequencer. The information reported here will be useful to understand the biology of lactation in sheep, providing also an opportunity to characterize their different patterns on milk production aptitude.

## Background & Summary

The development of high-throughput whole-transcriptome sequencing technologies, ie, RNA-Seq, has induced a revolutionary impact on transcriptome analysis. RNA-Seq technology enables the generation of extensive transcriptome information providing advantages over previous microarray analyses due to its wide dynamic range and its capability to exhaustively quantify the studied samples transcripts and not only the targets present on the array^1^. Furthermore, the high sequencing depth and coverage of this technology additionally provides structural information including alternative splice forms and transcriptome single nucleotide polymorphism^2^.

In recent years, RNA-Seq technology has been applied to the study of lactating mammary gland in several species^3–8^. The knowledge of the transcriptome profiling of the lactating mammary gland is of special interest since it allows the characterization of the genes implicated in the biology of lactation and the physiological and metabolic changes occurring in the mammary gland during this period. Besides, in dairy livestock the knowledge of the transcripts expressed in lactating mammary gland enhances our understanding on the genes underlying dairy traits, including milk yield and composition, milk technological properties, lactation persistency, etc.

The principal aim of this study was to gain a better understanding of the sheep lactating mammary gland and to compare the mammary gland transcriptome of two sheep breeds with different dairy production characteristics, Spanish Churra and Assaf. To that end a total of eight healthy animals were selected to be included in the experiment, four Assaf and four Churra ewes. These two breeds were chosen as they are considered as two of the principal dairy sheep breeds farmed in Spain. Churra is a Spanish autochthonous breed, characterized by its rusticity^9^. Assaf is a more specialized dairy sheep developed as a crossbred between Awassi (5/8) and Milschchaf (3/8) breeds^9^. Lactation is normalized to 120 days in Churra and 150 days in Assaf. The Assaf milk yield (400 kg) is more than double of the milk yield in Churra (117 kg), although Assaf milk has lower fat (6.65 versus 7.01) and protein contents (5.40 versus 5.79) (http://www.magrama.gob.es/es/). In general, milk from Churra sheep shows better characteristics for the manufacturing of mature dairy products^10^.

**Figure 1:**
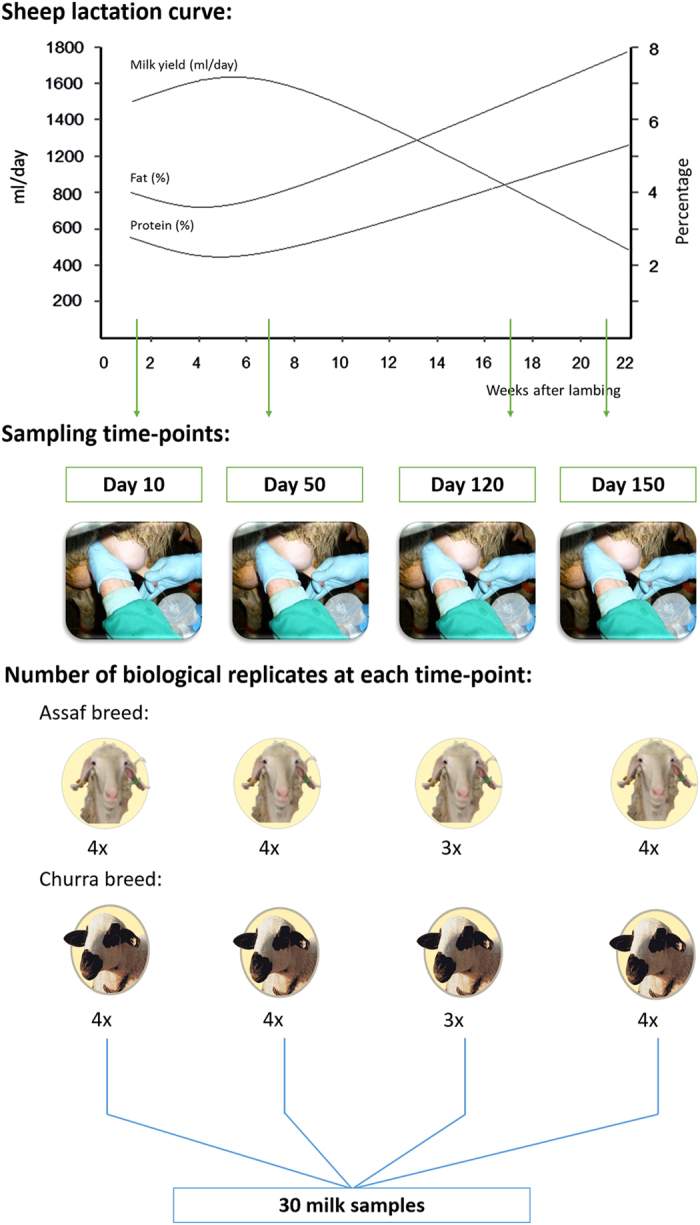
Overview of the study design. Considering a generic sheep lactation curve (provided in the top of the figure), the sampling points of this experiment, represented with green arrows in the graphic, were established to cover the different physiological stages of the mammary gland across the lactation curve. Milk samples were collected on days 10 (D10), 50 (D50), 120 (D120) and 150 (D150) after lambing. A total of eight healthy Assaf and Churra sheep were selected to be included in the experiment. At each sampling time point, four Assaf and four Churra ewes were milked to obtain milk somatic cells as source of RNA. Based on the quality scores of the extracted RNA samples for each breed, a total of 30 samples were sequenced: samples from four animals for the time points D10, D50 and D150, whereas three biological replicates were sequenced for D120.

For all the animals included in the present experiment, milk samples were collected on days 10 (D10), 50 (D50), 120 (D120) and 150 (D150) after lambing (Table 1, Fig. 1). These sampling points were established to cover the different physiological stages of the mammary gland across the complete lactation (Fig. 1). All the collected milk samples were later processed to extract RNA from the milk somatic cells (MSCs). MSCs contain heterogeneous populations of cells^11,12^. The proportions of these cellular populations in sheep milk were review by Li *et al.*^11^ Among MSCs, mammary epithelial cells (MECs) are the cells that produce milk and are shed from the mammary epithelium during lactation. In ruminants, this type of cell is often detected below 15%^11^. Specifically, in ewe’s milk, MECs represent a minority of total MSCs content, 2 to 3%; reviewed by Herve *et al.*^12^ Nevertheless, this value should be used as a rough estimation since, for dairy cows, where milk cells subpopulations have been more studied than in sheep, the estimation of MECs concentration in milk has a wide range of variation depending, among other factors, on the counting method used (reviewed by Herve *et al.*^12^). For one of the studied breeds, Churra, a study on the variation in the total number and proportions of milk cells types according to total cell counts has been reported^13^. In this study, MECs were included in ‘other cells’ and the proportion range between 10 and 18% of total MSCs for hand milking ewes with total somatic cells counts below 200,000 cells ml^−1^ (ref. 13).

For our study, MSCs cells were selected as RNA source based on cattle studies that have shown MSCs as a representative source of the RNA expressed in the mammary gland tissue^14^, showing, for the gene expression levels, high average correlations with mammary gland biopsy (r=0.95) and laser microdissected mammary epithelial cells (r=0.87)^14^. Moreover, MSCs provide a more accessible method compared with invasive approaches, such as mammary gland biopsies. This later point is of relevance when undertaking dynamic studies requiring several sampling time points for the same animal^15^. Regarding the potential variation of MSCs during the lactation cycle, advancing lactation has been associated to an increase of MSCs in milk^16^. This increment is firstly due to a concentration effect as a result of the reduction of milk yield that occurs after the lactation peak. In addition, rises in MSCs have generally been associated to an increase of polymorphonuclear cells^13,17^. However, it has been demonstrated that advancing lactation has also a stimulatory effect on MECs exfoliation process^12^, thus, there is also an increase of MECs towards late lactation^12^.

The RNA-Seq profiling dataset was generated on high-quality total RNA on an Illumina HiSeq 2000 platform (Table 2). This approach generated a total of 1,116 million paired-end reads from the transcriptome sequencing of the 30 milk samples. All samples had a suitable level of real quality, a high mapping rate (Table 2, Technical validation) and no contamination was found through the alignment against the *Escherichia coli* genome. The highly expression of genes codifying for major milk proteins in all the stages of lactation analysed supported that the gene expression profile of MSCs are representative from lactating mammary gland. To the best of our knowledge, this dataset (GE) represents the largest public RNA-Seq longitudinal dataset on sheep lactating mammary gland. In the related work published on Scientific Reports we performed an in depth analysis of these data, providing the first integrated overview on sheep milk gene expression across lactation^18^. The dataset reported in this data descriptor may be helpful for future studies examining the biology of sheep lactation.

## Methods

### Power calculation

The online tool Scotty (http://scotty.genetics.utah.edu/) was used in the design of the RNA-Seq experiment. This tool enables the calculation of the optimal sequencing depth and the number of replicates needed per condition to plan RNA-Seq experiments with adequate power to detect differential expression. The power calculations on Scotty (http://scotty.genetics.utah.edu/) require to upload a prototype dataset and to fix several experimental constraints for power optimization. As prototype dataset, we used our own pilot RNA-Seq data obtained from MSCs from four sheep per breed. To estimate the power based on our pilot dataset we set the following parameters: a cost per replicate of 50 US Dollars (USD), a cost per million reads aligned to genes of 150 USD, an alignment rate of the 85%, a maximum of 10 replicates per condition, a read depth between 10 and 40 millions of reads, a maximum cost of the experiment of 100,000 USD, a 50% of differential expressed genes detected with a fold change of 2 and a *P*-value of 0.01 and a minimum of 30% of genes with at least 50% of maximum power.

### Animals and sampling

This description of the selected animals and the sampling method is extended from descriptions in the related research manuscript^18^. The trial was initiated with thirteen non-related sheep, eight Assaf and five Churra ewes. The animals belong to the commercial farm of the University of León (Spain). These sheep were kept in free stall housing, fed with the same rations and did not endure any water restriction. Animals were machine milked twice a day: at 8 a.m. and 5 p.m. For all these ewes, lambing took place between November 11th, 2012, and December 11th, 2012. All the selected ewes were between their fourth and sixth parities. During the course of the lactation, official monthly test-day records for milk yield, somatic cell count (SCC) and fat, protein and total solids contents were performed by the corresponding breeders´ association. According to the SCC records, animals with high level of SCC (> 250,000 SCC per milliliter^19^), which is associated with subclinical mastitis, were discarded from the experiment (three Assaf and one Churra ewes). Finally, a total of eight healthy sheep were selected to be included in the experiment, four Assaf and four Churra ewes. The lactation phenotypic values of the ewes selected for this study are shown in Table 3.

Trying to cover the evolution of the mammary gland transcriptome across lactation, milk samples were collected on days 10 (D10), 50 (D50), 120 (D120) and 150 (D150) after lambing. D10 is the first day of lactation considered to be totally free of colostrum; it is also the day considered as starting point in the normalized lactation for both breeds. D50 is a time point close to the lactation peak in both breeds, although Churra shows an earlier peak (range days 35–45 (ref. 15)) than Assaf sheep (range days 45–55 (ref. 16)). The D120 and D150 sampling points correspond to the end of the normalized lactation in Churra and Assaf, respectively. Hence, whereas for Churra D120 is close to the final lactation point, for Assaf this time point corresponds to a transition stage from the lactation peak to the final lactation point (D150). For each sampled animal and lactation point, at least four milk samples of 50 ml were collected; two of them were obtained on the exact sampling day whereas two additional samples were collected the previous or the following day to ensure RNA source material for each desired sampling.

With the aim of maximizing the number of somatic cells present in milk, the sample collection was performed one hour after the 8 a.m. routine milking and ten minutes after the injection of 5 IU of Oxitocine Facilpart (Syva, León, Spain). The time of milk sample collection was chosen based on previous studies that indicate that one hour after milking is the diurnal time point with the highest concentration of MSCs^20^. Oxytocin was just administrated on sampling days to avoid any effect on milk composition and with the aim of stimulating the mechanical effect of myoepithelial contraction and thus the flattering of the alveolar lumen that causes the release of the residual post-milking milk which has a higher concentration of exfoliated MECs^21^. All protocols involving animals were approved by the Animal Welfare Committee of the University of Leon, Spain, following proceedings described in Spanish and EU legislations (Law 32/2007, R.D. 1201/2005, and Council Directive 2010/63/EU). The animals used in this study were handled in strict accordance with good clinical practices and all efforts were made to minimize suffering.

To ensure RNA purification of high yield and quality, we used the following protocol during the sampling process. Before sampling, the collection milk containers were cleaned with RNaseZap (Ambion, Austin, TX, USA) and autoclaved. In the farm, udder cleaning was performed with special care: first, the udders were cleaned with water and soap; then, they were disinfected with povidone iodine; and finally the nipples were cleaned with RNAseZap (Ambion, Austin, TX, USA). Milk samples were collected from both mammary glands. A sterile gauze was used to cover the collection container during milk collection to minimize the risk of sample contamination. After collection the milk was transferred to 50 ml RNAse-free tubes. Samples were maintained at 4 °C during their transport from the farm to the laboratory where they were immediately processed.

### RNA extraction

This description of RNA extraction is extended from the protocol described in the related research manuscript^18^. Samples of approximately 50 ml of milk were used for the RNA extraction. The pellet of MSCs was obtained as described by Wickramasinghe *et al.*^3^ with some modifications. The cells were pelleted by centrifugation, at 540×*g* for 10 minutes at 4 °C, and in the presence of a final concentration of 0.5 mM of EDTA to eliminate casein and fat globules. After centrifugation, the supernatant was discarded. During this step, a fatty layer frequently appeared on the top of the tube. To remove it, a sterile pipette tip was introduced to separate this fatty layer from the tube walls. Then, the cell pellet was washed in 10 ml of PBS (pH 7.2) with 0.5 mM EDTA and centrifuged at 540×*g* in 15 ml RNAse free sterile tubes for 10 min at 4 °C. The last step was repeated until the fatty layer was minimized (usually twice). Once the pellet was clean, it was resuspended in 500 μl of Trizol (Invitrogen, Carlsbad, CA, USA) and homogenized by vortexing. Immediately after that, the following steps were performed: first, the homogenized sample was incubated for 15 min at room temperature to permit the complete dissociation of the nucleoprotein complex. After incubation, 100 μl of chloroform were added. Then, the sample was shaken vigorously by hand for 15 s, incubated 15 min at room temperature and centrifuged at 12,000×*g* for 15 min at 4 °C. After centrifugation, the upper aqueous phase of the sample was taken and placed in a new tube where 250 μ of isopropanol were added. The sample was then incubated for ten minutes at room temperature and centrifuged at 12 000×*g* for 15 min at 4 °C. After centrifugation, the supernatant was removed from the tube, leaving only the RNA pellet. The RNA pellet was washed with 0.5 ml of ethanol. Then, the sample was vortexed briefly and the tube was centrifuged at 7,500×*g* for 5 min at 4 °C. After the ethanol was discarded, the sample was dried for seven minutes at room temperature. To elute the sample 150 μl of DEPC water with DNAse (0.2 μl in 100 μl) was added and then, it was incubated for 10 min at 55 °C. Once diluted, the sample was stored at −80 °C.

### RNA sequencing

This description on RNA sequencing is extended from the description presented in the related research manuscript^18^. The Agilent 2100 Bioanalyzer device (Agilent Technologies, Santa Clara, CA, USA) was used to assess the integrity of the RNA. Based on the quality scores of the extracted RNA samples a total of 30 RNA samples were sequenced. For each breed, samples from four animals were sequenced for time points D10, D50 and D150, whereas three biological replicates were sequenced for D120. The RNA integrity value (RIN) of the samples selected to be sequenced ranged between 7.1 and 9 (Table 2). Paired-end libraries with fragments of 300 bp were prepared using the True-Seq RNA-Seq sample preparation Kit v2 (Illumina, San Diego, CA, USA). The fragments were sequenced on an Illumina Hi-Seq 2000 sequencer (Fasteris SA, Plan-les-Ouates, Switzerland), according to the manufacturer’s instructions at CNAG (Centro Nacional de Análisis Genómico, Barcelona, Spain). For each library, between 35–45 million paired-end 75 bp reads were generated during the sequencing run (Table 2). The Fastq files generated were deposited in the Gene Expression Omnibus (GEO) database under the accession number GSE74825.

### RNA-Seq data analysis

The read quality of the RNA-seq libraries was evaluated using FastQC (http://www.bioinformatics.babraham.ac.uk/projects/fastqc/). Reads were mapped against the ovine genome assembly v.3.1. (Oar_v3.1) using the STAR aligner (v.2.3.1y)^22^. The data was also tested for contamination on the *Escherichia coli* genome using BWA^23^. Cuffquant and Cuffnorm packages from Cufflinks^24^ were used to compare gene expression levels within the same sample. Gene abundances were normalized by library and gene length by calculating Fragments Per Kilobase Of Exon Per Million Fragments Mapped (FPKM) using the Ensembl annotated genes (Oar_v3.1) as a reference.

The Cufflinks and Cuffmerge tools from the Cufflinks package^24^ were used to create a ‘transcripts.gtf’ file to be used as reference in our assembly. The aim of the assembly was producing a new annotation reference including novel genes and transcripts to be used in the downstream differential expression analyses. The Cufflinks option ‘−g’ followed by the available gtf file from the Oar_v3.1 reference sequence was used to guide the assembly but without excluding new genes. Cuffmerge was used to filter genes with low or no expression from our reference gtf file. To compare the expression levels of genes across samples, raw counts for the genes and transcripts were obtained using SigCufflinks (available at http://www.sigenae.org) using de ‘-G’ option of SigCufflinks to guide the alignment but excluding new genes. SigCufflinks is a modified version of the cufflinks code that provides raw read counts per gene and transcript, by using the sorted bam file from the alignment and the reference gtf file created in the assembly. The output file form Sigcufflinks containing raw counts per gene was deposited in the Gene Expression Omnibus (GEO) database under the accession number GSE74825. Downstream differential expression analyses were performed with edgeR^25^ and DESeq2^26^ R packages, as indicated in the related research manuscript^18^.

## Data Records

The raw fastq files for the RNA-seq libraries were deposited at the Gene Expression Omnibus (GEO) database under the accession number GSE74825 (Data Citation 1). The processing of all fastq samples is summarized in Tables 1 and 2. The output file from the quantification of transcripts by Sigcufflinks is also deposited in the Gene Expression Omnibus (GEO) under the same accession number GSE74825. It contains all the genes identified in the assembly and the raw counts per gene for each sample.

## Technical Validation

### Power calculations

**Figure 2:**
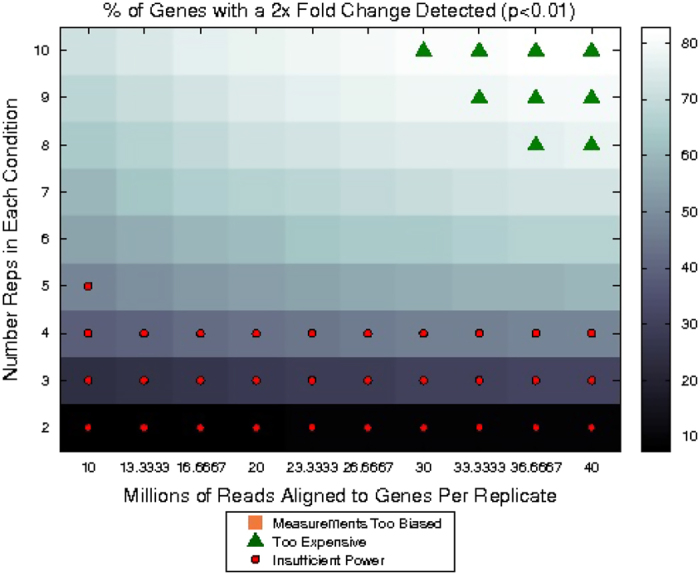
Chart showing the power achieved in each of the experimental configurations tested by Scotty (http://scotty.genetics.utah.edu/). Millions of reads aligned to genes per replicate are represented in the X-axis, whereas the number of replicates in each condition is represented in the Y-axis. The percentage of genes detected with a 2x Fold Change are coloured as indicated in the bar placed at the right of the grid. Experiments that do not conform the fixed constraints are filled as indicated in the legend.

The results for the power estimates achieved in each experiment configuration tested with Scotty (http://scotty.genetics.utah.edu/) are described in Supplementary File 1 and summarized in Fig. 2. The least expensive experiment that has enough power to perform a differential expression analysis according to the settings fixed was sequencing six replicates to a depth of 10 million aligned reads per replicate. The most powerful experiment that matches our criteria was sequencing 10 replicates to a depth of 26.67 million reads to genes. According to these results, the animals available to perform the experiment and the nature of the lactating mammary gland transcriptome (mostly enriched in transcripts codifying for major milk proteins), we finally decided to sequenced the MSCs RNA samples from eight replicates (four Churra and four Assaf) at each of the lactation time-points selected for the study (with the exception of D120 for which only 6 replicates were sequenced) to an average depth of 35 million reads.

### Quality control of RNA

Total RNA integrity was assessed by the RNA Integrity Number (RIN) algorithm calculated by the Agilent Bioanalyzer software. The Agilent Bioanalyzer RIN scores are listed in Table 2. All the total RNA samples used for this RNA-seq study had a RIN score above 7 showing the high integrity of the samples used.

### Quality validation and analysis of RNA-seq data

A total of 30 RNA libraries were sequenced to a depth between 23–46 million paired-end reads among which about 88.10% of the reads mapped to unique locations in the ovine genome assembly (Oar_v3.1) (Table 2). No contamination was found in the alignment against the *Escherichia coli* genome.

**Figure 3:**
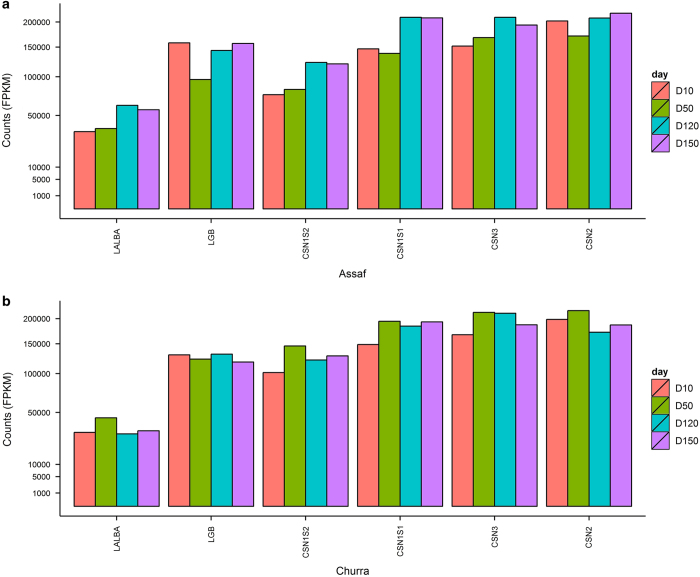
Bar graph for the six highly expressed genes identified across lactation in the milk samples of the studied two sheep breeds (Churra and Assaf). FPKM values are represented in the X-axis, whereas the gene names are indicated in the Y-axis. A colour code is used to represent the four time points studied: day 10 (D10), 50 (D50), 120 (D120) and 150 (D150). (**a**) Highly expressed genes in Assaf. (**b**) Highly expressed genes in Churra.

In order to validate the quality of the RNA-seq libraries as representative from lactating mammary gland, we evaluated the profile of the highly expressed genes identified for our samples. As expected, the genes with the highest FPKM values for both sheep breeds and at the four studied lactation time points are *CSN2* (β-casein), *CSN3* (κ-casein), *ENSOARG00000005099* (*LGB*, β-lactoglobulin), *CSN1S2* (casein-α-S2), *CSN1S1* (α-S1-casein) and *LALBA* (α-lactalbumin) (Fig. 3), accumulating at approximately the 65% of the total gene FPKM reads at each of the analysed time points. These highly expressed genes encode four caseins and two whey proteins, principal components of milk, which encompass the 5.5% of total milk composition in sheep. Thus, although it has been remarked that MECs are a minor proportion of total MSCs in sheep, the highly expression of genes codifying for major milk proteins in all the stages of lactation demonstrated that the MSCs transcriptome is principally dominated for the expression of MECs, probably due to the high transcription activity of these cells during lactation.

**Figure 4:**
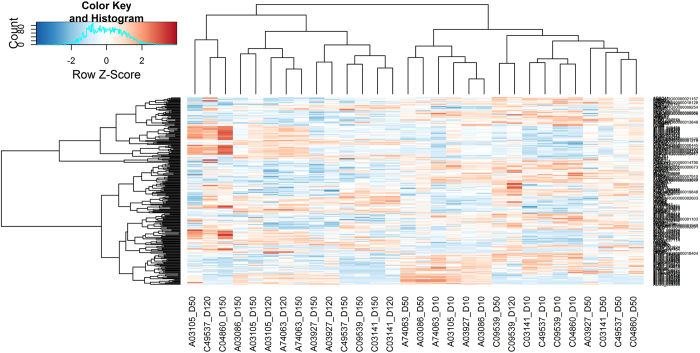
Heatmap and hierarchical clustering of the differentially expressed genes (DEGs) across lactation and between the Assaf and Churra sheep breeds. Heatmap display of supervised hierarchical clustering of the DEGs identified across the four considered time points of the sheep lactation (D10, D50, D120, D150) and between both breeds. The genes are displayed in rows and the normalized counts per sample are displayed in columns. Each column represents a sample; sample names indicate the corresponding breed (A=Assaf; C=Churra) and day of sampling (D10, D50, D120, D150). A colour code indicates up-regulated (orange) and down-regulated (blue) expression levels.

The principal aim of this study was the dynamic analysis of the sheep mammary gland transcriptome through MSCs. For the analysis we selected samples from two sheep breeds, Assaf and Churra. Both are dairy breeds differing on milk production traits, mainly in terms of milk yield and milk composition (explained in Background & Summary). However, it is necessary to clarify that this experimental design does not involve the analysis of extreme phenotypes and therefore completely differs from a case-control study. This would explain the high correlation observed between all the samples analysed (*r*^*2*^>0.86). By plotting a heatmap using hierarchical clustering with the genes found as differentially expressed in common with the edgeR^25^ (FDR<0.05) and DESeq2 (ref. 26) (p_adj_-value < 0.05) packages between all the time points analysed and between both breeds (Fig. 4), it can be observed that the samples are mainly clustered in two major groups, one corresponding to the D10 and D50 time points (related to the initial stages of lactation for both breeds) and the other corresponding to D120 and D150 time points (associated with the late stages of lactation). These observations confirm that the considered set of samples is highly representative from initial and final stages of lactation in sheep, although some differences have also been found between breeds (see the related research manuscript^18^). As normal samples, with no evidence of disease or particular phenotype, these samples would be a useful complement for other studies focused on the analysis of the sheep mammary gland transcriptome through RNA-Seq.

## Usage Notes

The RNA-Seq fastq files could be aligned using publicly splice-aware software solutions like TopHat2 (ref. 27) or STAR^22^. As reference genome we have used the ovine genome assembly (Oar_v3.1) downloaded from Ensembl database (http://www.ensembl.org/Ovis_aries/Info/Index). Cufflinks package^24^ could be used to perform the assembly, quantification and differential expression analysis but also other publicly software combinations could be used for quantification and differential expression analysis: e.g. SigCufflinks (available at http://www.sigenae.org) or HTSeq^28^ for quantification, combined with edgeR^25^ or DESeq2 (ref. 26) for the differential expression analyses. Based on power estimations (Fig. 2) we recommend to use at least 5 replicates per condition to perform differential expression analysis. Functional analysis of the RNA-Seq differential expressed genes could be performed with several software solutions such as Babelomics^29^, WebGestalt^30^ or QIAGEN’s Ingenuity Pathway Analysis (IPA, QIAGEN Redwood City, www.qiagen.com/ingenuity)

## Additional Information

**How to cite**: Suárez-Vega, A. *et al.* Comprehensive RNA-Seq profiling to evaluate lactating sheep mammary gland transcriptome. *Sci. Data* 3:160051 doi: 10.1038/sdata.2016.51 (2016).

## Supplementary Material



Supplementary File 1

## Figures and Tables

**Table 1:** Characterization and identification of the samples included in the experimental design described here.

**Subjects**	**Source**	**Sample**	**Characteristics**		**Protocol 1**	**Protocol 2**	**Protocol 3**	**Data**
			**Breed**	**Day of lactation**				
Sheep_1	Sheep milk	A03086_D10	Assaf	Day 10	Milk somatic cells isolation	RNA extraction	RNA-seq	GSM1936059
Sheep_2	Sheep milk	A03105_D10	Assaf	Day 10	Milk somatic cells isolation	RNA extraction	RNA-seq	GSM1936060
Sheep_3	Sheep milk	A03927_D10	Assaf	Day 10	Milk somatic cells isolation	RNA extraction	RNA-seq	GSM1936061
Sheep_4	Sheep milk	A74063_D10	Assaf	Day 10	Milk somatic cells isolation	RNA extraction	RNA-seq	GSM1936062
Sheep_5	Sheep milk	C03141_D10	Churra	Day 10	Milk somatic cells isolation	RNA extraction	RNA-seq	GSM1936063
Sheep_6	Sheep milk	C04860_D10	Churra	Day 10	Milk somatic cells isolation	RNA extraction	RNA-seq	GSM1936064
Sheep_7	Sheep milk	C49537_D10	Churra	Day 10	Milk somatic cells isolation	RNA extraction	RNA-seq	GSM1936065
Sheep_8	Sheep milk	C09539_D10	Churra	Day 10	Milk somatic cells isolation	RNA extraction	RNA-seq	GSM1936066
Sheep_1	Sheep milk	A03086_D50	Assaf	Day 50	Milk somatic cells isolation	RNA extraction	RNA-seq	GSM1936067
Sheep_2	Sheep milk	A03105_D50	Assaf	Day 50	Milk somatic cells isolation	RNA extraction	RNA-seq	GSM1936068
Sheep_3	Sheep milk	A03927_D50	Assaf	Day 50	Milk somatic cells isolation	RNA extraction	RNA-seq	GSM1936069
Sheep_4	Sheep milk	A74063_D50	Assaf	Day 50	Milk somatic cells isolation	RNA extraction	RNA-seq	GSM1936070
Sheep_5	Sheep milk	C03141_D50	Churra	Day 50	Milk somatic cells isolation	RNA extraction	RNA-seq	GSM1936071
Sheep_6	Sheep milk	C04860_D50	Churra	Day 50	Milk somatic cells isolation	RNA extraction	RNA-seq	GSM1936072
Sheep_7	Sheep milk	C49537_D50	Churra	Day 50	Milk somatic cells isolation	RNA extraction	RNA-seq	GSM1936074
Sheep_8	Sheep milk	C09539_D50	Churra	Day 50	Milk somatic cells isolation	RNA extraction	RNA-seq	GSM1936073
Sheep_2	Sheep milk	A03105_D120	Assaf	Day 120	Milk somatic cells isolation	RNA extraction	RNA-seq	GSM1936075
Sheep_3	Sheep milk	A03927_D120	Assaf	Day 120	Milk somatic cells isolation	RNA extraction	RNA-seq	GSM1936076
Sheep_4	Sheep milk	A74063_D120	Assaf	Day 120	Milk somatic cells isolation	RNA extraction	RNA-seq	GSM1936077
Sheep_5	Sheep milk	C03141_D120	Churra	Day 120	Milk somatic cells isolation	RNA extraction	RNA-seq	GSM1936078
Sheep_7	Sheep milk	C49537_D120	Churra	Day 120	Milk somatic cells isolation	RNA extraction	RNA-seq	GSM1936080
Sheep_8	Sheep milk	C09539_D120	Churra	Day 120	Milk somatic cells isolation	RNA extraction	RNA-seq	GSM1936079
Sheep_1	Sheep milk	A03086_D150	Assaf	Day 150	Milk somatic cells isolation	RNA extraction	RNA-seq	GSM1936081
Sheep_2	Sheep milk	A03105_D150	Assaf	Day 150	Milk somatic cells isolation	RNA extraction	RNA-seq	GSM1936082
Sheep_3	Sheep milk	A03927_D150	Assaf	Day 150	Milk somatic cells isolation	RNA extraction	RNA-seq	GSM1936083
Sheep_4	Sheep milk	A74063_D150	Assaf	Day 150	Milk somatic cells isolation	RNA extraction	RNA-seq	GSM1936084
Sheep_5	Sheep milk	C03141_D150	Churra	Day 150	Milk somatic cells isolation	RNA extraction	RNA-seq	GSM1936085
Sheep_6	Sheep milk	C04860_D150	Churra	Day 150	Milk somatic cells isolation	RNA extraction	RNA-seq	GSM1936086
Sheep_7	Sheep milk	C49537_D150	Churra	Day 150	Milk somatic cells isolation	RNA extraction	RNA-seq	GSM1936088
Sheep_8	Sheep milk	C09539_D150	Churra	Day 150	Milk somatic cells isolation	RNA extraction	RNA-seq	GSM1936087

**Table 2:** Sample quality and read statistics.

**Sample**	**Total RNA QC**		**Sequencer**	**Read length (bp)**	**Million read-pairs (or reads)**	**Uniquely mapped reads (%)**
	**ng**	**RIN**				
A03086_D10	5.76	8.0	illumina HiSeq 2000	2×75	36.74	84.5
A03105_D10	5.04	8.3	illumina HiSeq 2000	2×75	33.79	86.7
A03927_D10	5.74	7.8	illumina HiSeq 2000	2×75	31.89	86.1
A74063_D10	2.65	7.7	illumina HiSeq 2000	2×75	42.03	87.4
C03141_D10	4.69	7.1	illumina HiSeq 2000	2×75	30.69	87.8
C04860_D10	6.58	7.7	illumina HiSeq 2000	2×75	28.70	84.9
C49537_D10	14.31	8.5	illumina HiSeq 2000	2×75	36.96	88.3
C09539_D10	4.76	8.0	illumina HiSeq 2000	2×75	23.93	86.3
A03086_D50	2.55	7.5	illumina HiSeq 2000	2×75	41.40	88.5
A03105_D50	20.65	8.5	illumina HiSeq 2000	2×75	43.29	88.8
A03927_D50	3.55	7.2	illumina HiSeq 2000	2×75	30.59	84.9
A74063_D50	5.51	7.4	illumina HiSeq 2000	2×75	35.69	89.4
C03141_D50	4.44	8.1	illumina HiSeq 2000	2×75	29.88	86.9
C04860_D50	9.67	7.9	illumina HiSeq 2000	2×75	46.95	90.1
C49537_D50	3.52	7.8	illumina HiSeq 2000	2×75	43.25	88.0
C09539_D50	4.96	9	illumina HiSeq 2000	2×75	46.81	90.3
A03105_D120	10.86	8.2	illumina HiSeq 2000	2×75	42.49	88.1
A03927_D120	3.20	8.9	illumina HiSeq 2000	2×75	39.68	87.8
A74063_D120	2.74	8.5	illumina HiSeq 2000	2×75	36.84	89.6
C03141_D120	2.55	8.8	illumina HiSeq 2000	2×75	35.86	89.6
C49537_D120	4.41	8.9	illumina HiSeq 2000	2×75	34.35	87.3
C09539_D120	2.65	8.7	illumina HiSeq 2000	2×75	40.57	89.5
A03086_D150	4.95	8.7	illumina HiSeq 2000	2×75	38.14	88.6
A03105_D150	4.03	8.4	illumina HiSeq 2000	2×75	31.46	87.5
A03927_D150	3.03	8.6	illumina HiSeq 2000	2×75	41.54	89.3
A74063_D150	2.51	8.1	illumina HiSeq 2000	2×75	36.19	89.2
C03141_D150	5.26	8.6	illumina HiSeq 2000	2×75	32.06	89.6
C04860_D150	2.93	8	illumina HiSeq 2000	2×75	43.77	87.3
C49537_D150	4.44	8.7	illumina HiSeq 2000	2×75	44.05	90.9
C09539_D150	5.95	8.3	illumina HiSeq 2000	2×75	36.62	89.9

**Table 3:** Lactation phenotypic values of the ewes selected for the RNA-Seq analysis described here.

**Subjects**	**ID**	**Breed**	**Milk yield (Kg)**	**Somatic Cell Counts**	**Protein (%)**	**Fat (%)**	**Dry extract (%)**
Sheep_1	A03086	Assaf	402.14	215	5.33	5.43	14.99
Sheep_2	A03105	Assaf	477.38	66	4.43	5.87	16.78
Sheep_3	A03927	Assaf	213.32	117	5.8	7.74	16.39
Sheep_4	A74063	Assaf	270.95	178	4.79	5.98	16.44
Sheep_5	C03141	Churra	235.05	196	6.13	7.15	19.01
Sheep_6	C04860	Churra	131.21	100	6.3	7.68	19.68
Sheep_7	C49537	Churra	55.8	58	5.79	5.96	17.63
Sheep_8	C09539	Churra	94.73	140	5.76	7.50	18.86
