# Supplementary material for: Comprehensive RNA-Seq profiling to evaluate lactating sheep mammary gland transcriptome
Source: Sci Data. 2016 Jul 5;3:160051. doi: 10.1038/sdata.2016.51 (PMC4932878; doi:10.1038/sdata.2016.51)
Supplement: Supplementary File 1 [file sdata201651-s2.pdf]

Upload: sheep_milk_powercalculator_soloassaf.txt
File upload succeeded.

User Inputs Used in the Analysis

Control columns in pilot data: 4
Test columns in pilot data: 4
Cost per replicate, control: $50
Cost per replicate, test: $50
Cost per million reads: $150
Alignment Rate: 85%
Maximum cost of experiment: $100000
Percentage of genes detected: 50
At p value cutoff: 0.01
For the following true fold change: 2
Maximum percentage of genes with low-powered (biased) measurements: 30

Export To PDF

Summary of Findings

Scotty has tested 90 possible experimental designs.

The following experiments meet your criteria:

> Least expensive: 6 replicates sequenced to a depth of 10 million reads aligned to genes per replicate.
> Most powerful: 10 replicates sequenced to a depth of 26.6667 million reads aligned to genes per replicate.

The number of samples that is required is in part determined by how dispersed your biological replicates are. We measured the dispersion of your replicates:

> Control samples replicate dispersion: 0.2729
> Test samples replicate dispersion: 0.2361

The dispersion metric that Scotty uses is the mean overdispersion from Poisson. Many factor can affect how dispersed replicates are. For a general reference, most of the biological replicate pairs we examined had an overdipsersion between 0.2 and 0.4.

We measured the number of unique genes observed in you data (detected by at least one read in one or the samples) and estimated the number of genes that are expressed:

> Genes observed (Control): 18111
> Genes observed (Test): 17605

Power calculations (the % detected) are based on the number of observed genes.

Data Quality

Replication(?)

These are scatter plots which show how closely biological replicates in the same condition replicate.

Control Data

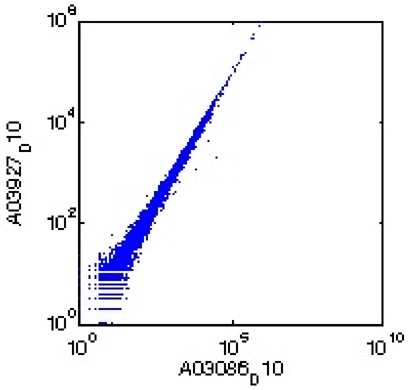

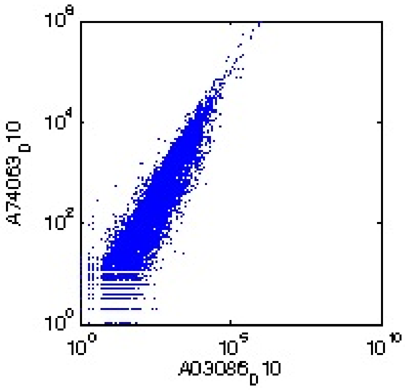

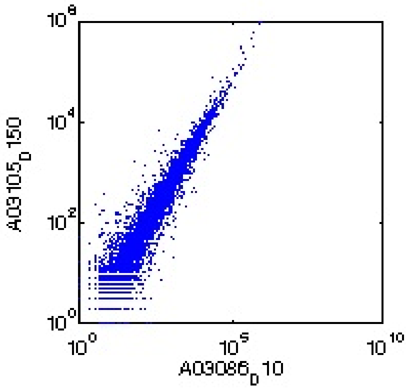

Test Data

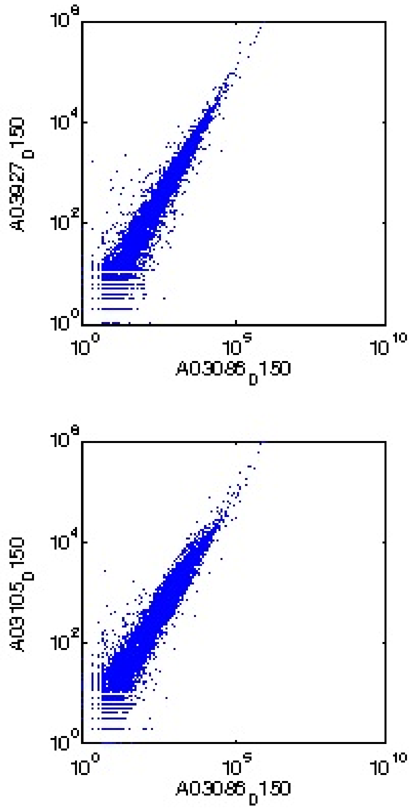

Control versus Test

This is a scatter plot of your combined control data versus your combined test data.

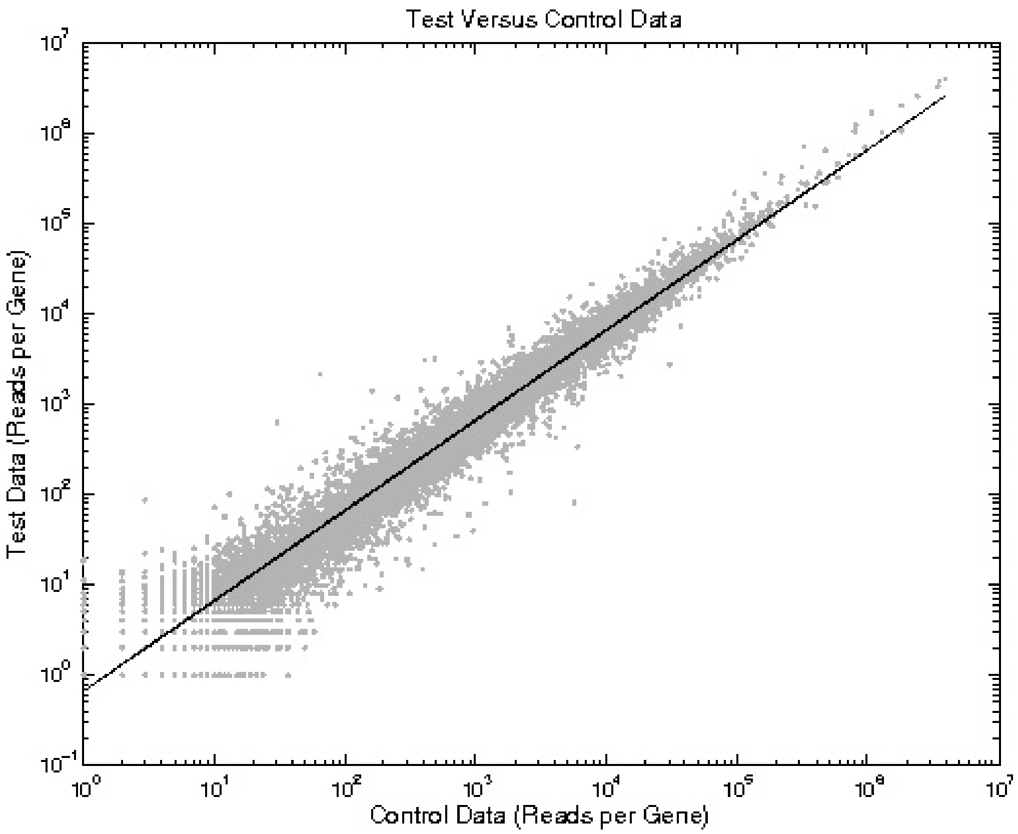

Clustering[(?)]

Scotty has clustered your data as a quality control check. Sample that are most similar should

group together, while samples that are different should be separated by a greater distance. In most, but not all cases, the control samples would be expected to cluster in a separate branch than the test samples. Hierarchical clustering was performed using the Spearman correlation as the distance metric. See example.

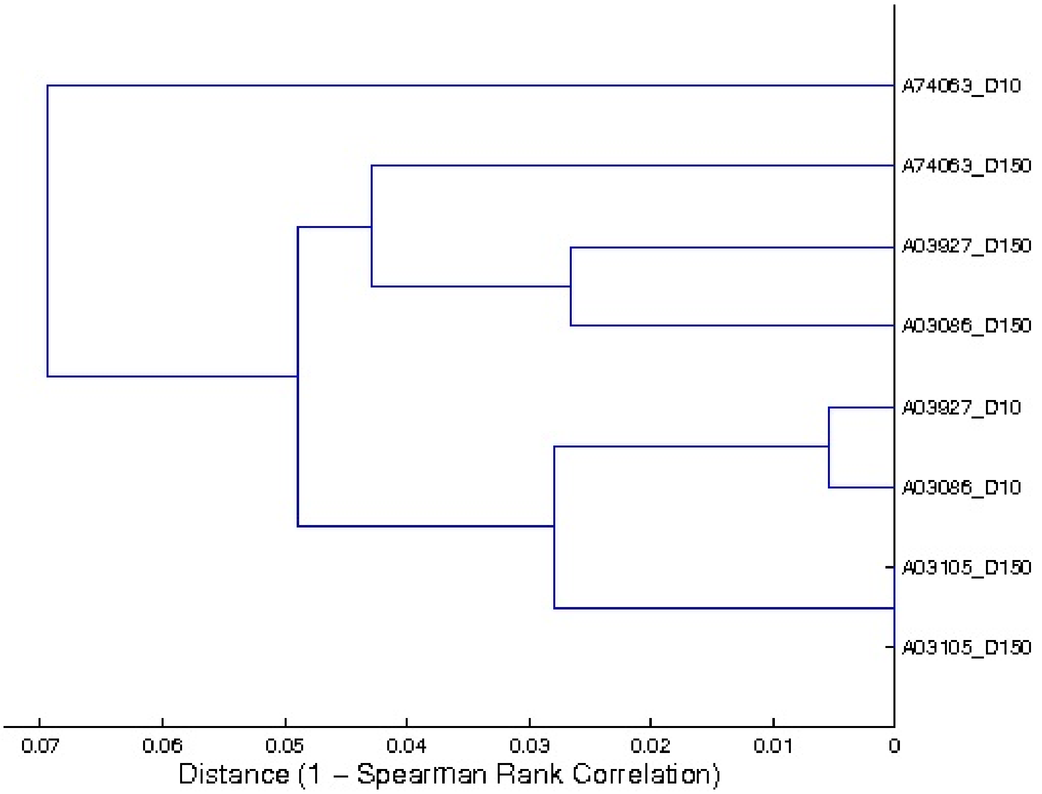

Genes Detected as a Function of Sequencing Depth

Sequencing more deeply will lead to the detection of a greater number of genes until saturation is reached. The X axis shows the sequencing depth (the number of reads aligned to genes). The Y axis shows the number of genes that will be detected by at least 10 reads at each sequencing depth.

This chart can be used to estimate how many genes are expressed at quantifiable levels, and how deeply samples should be sequenced to quantify a fixed number of genes. It can also be used to assess library complexity. Samples within a single condition should have similar curves. See example.

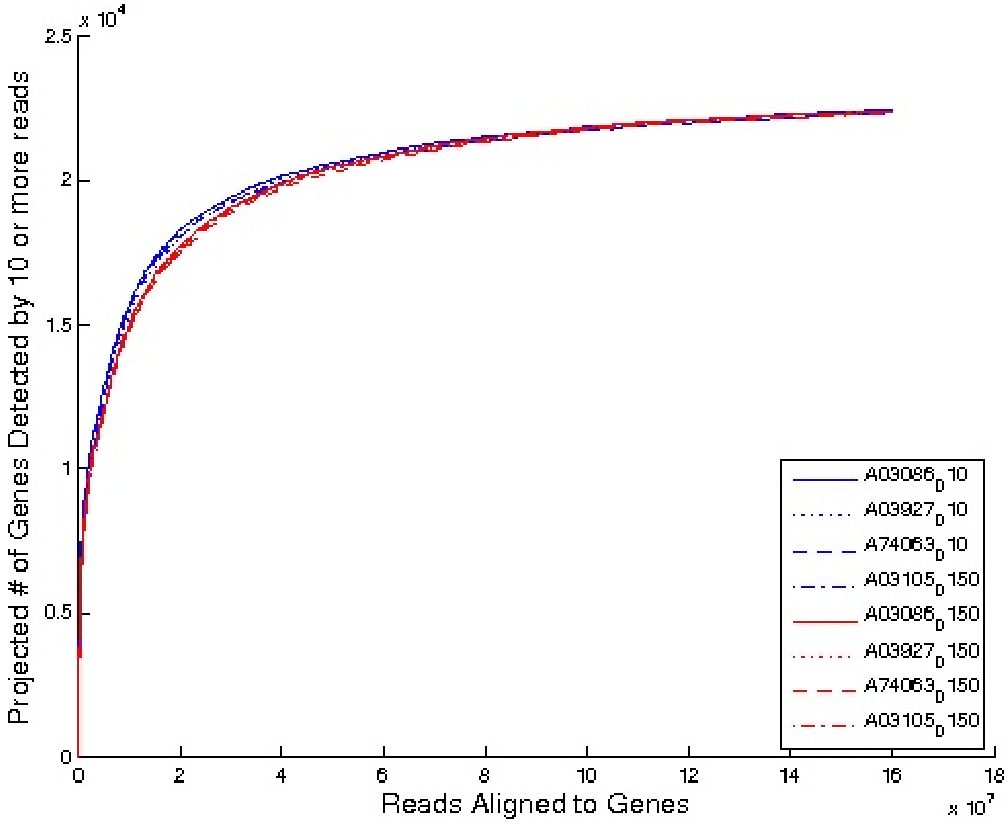

Optimization(?)

Scotty has tested several potential experimental configurations.
Results are presented in the following format:

Power By Experimental Configuration

This grid shows how much power each experimental configuration will have. Power is as measured by the percentage of genes which have a true 2X fold change that will be detected at p<=0.01 using a t-test.

Experimental configurations with the highest power will have the lightest boxes.
See example.

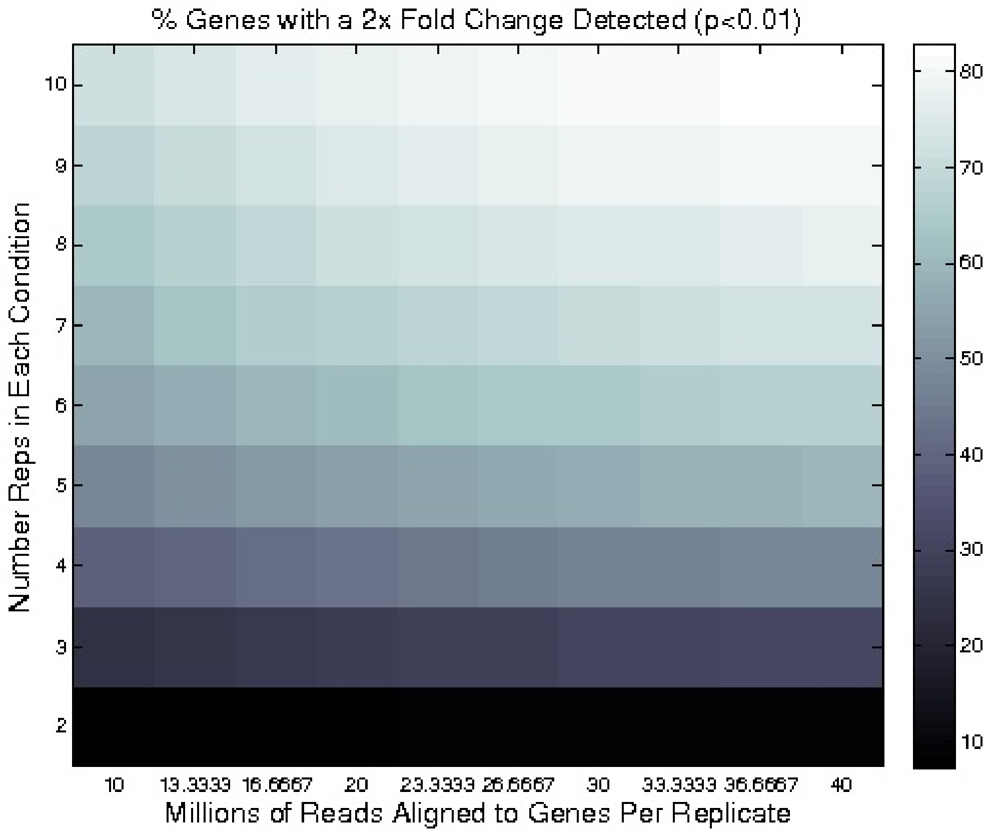

Cost By Experimental Configuration

This grid shows how much each experimental configuration will cost.

The most expensive configurations will have the brightest green boxes. [See example.](#)

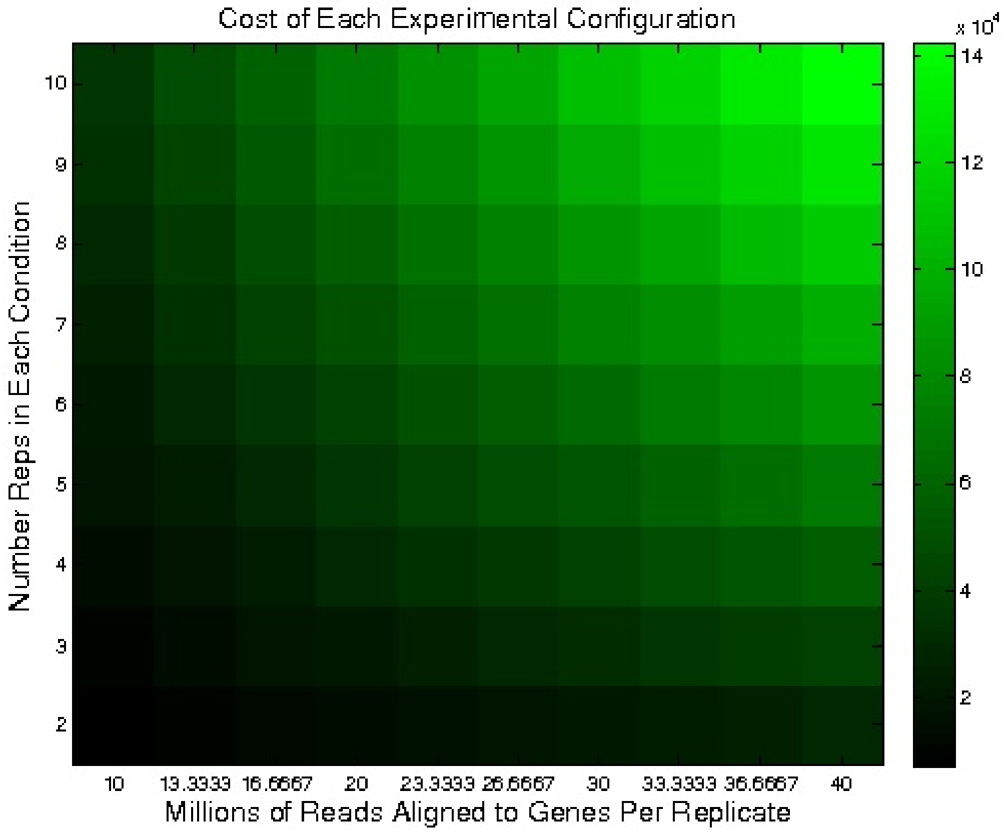

Measurment Biases By Experimental Configuration

The count nature of RNA Seq data introduces a certain degree of measurement bias into the experiment. This is caused because counts of 200 versus 100 are more likely to represent true differences in expression than counts of 2 versus 1. Therefore, genes measured with few reads are less likely to be detected as differentially expressed than genes with higher read counts, even if the fold change is the same.

This grid shows how much measurement bias is present in each experimental configuration.

As our metric of bias, we first define the "maximum power" as the percentage of genes that could be detected if measurements were made using continuous measurements (i.e. without Poisson counting noise). Measurement bias is then defined as the percentage of genes which are measured with at least 50% of the maximum power.

Experimental configurations with the highest bias will have the darkest boxes. A configuration with low bias may not be necessary for all experiments, but in some cases may result in a less complex downstream analysis. See example.

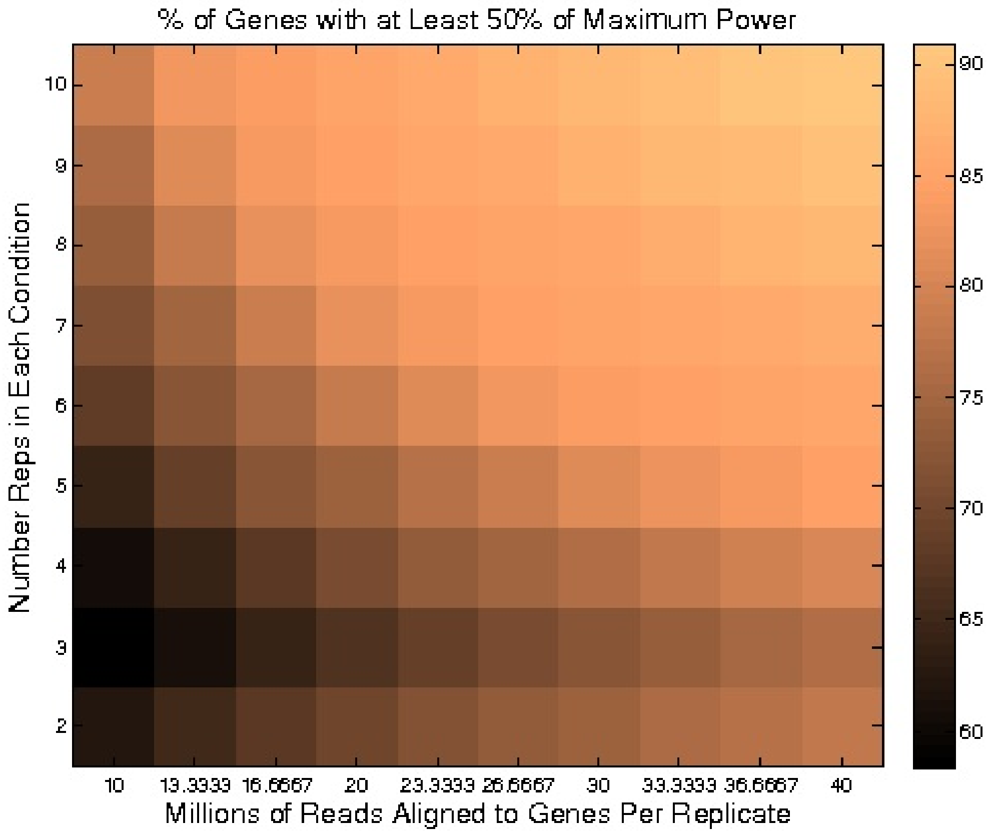

Excluded Experimental Configurations

This shows the power that will be achieved in each experimental configuration.
Filled in boxes are not allowed for the reasons stated in the key.
See example.

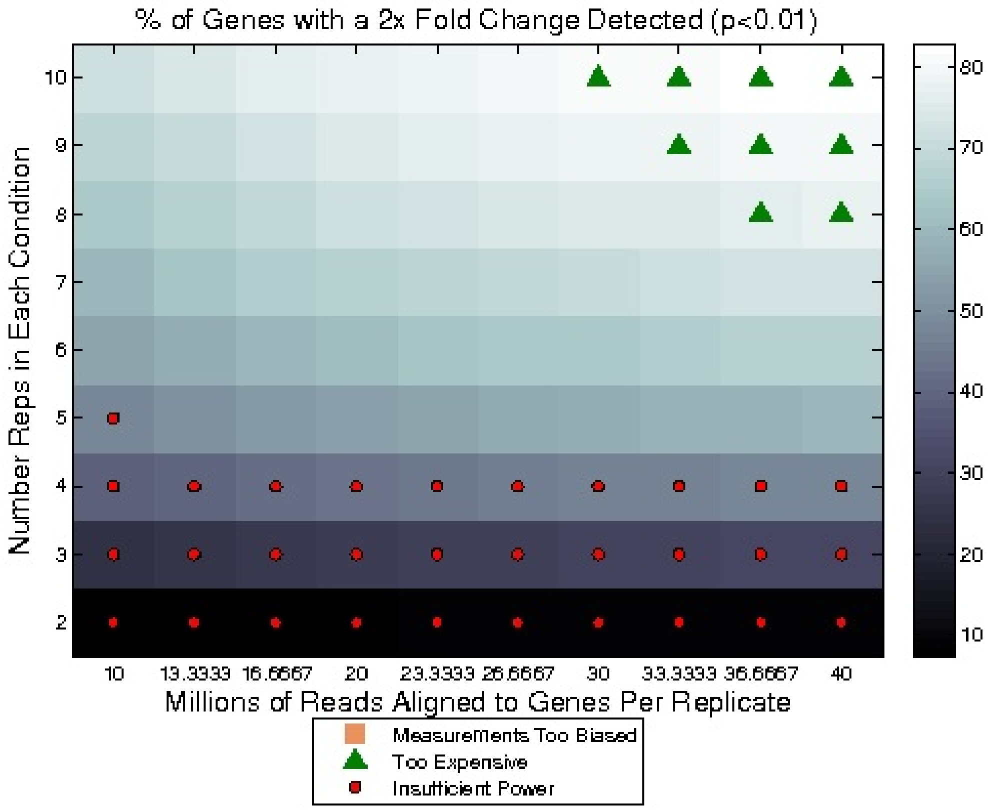

Allowed Experimental Configurations

This shows experimental configurations which are and are not allow under the user defined optimization parameters.
White boxes are allowed. Red boxes are not allowed . See example.

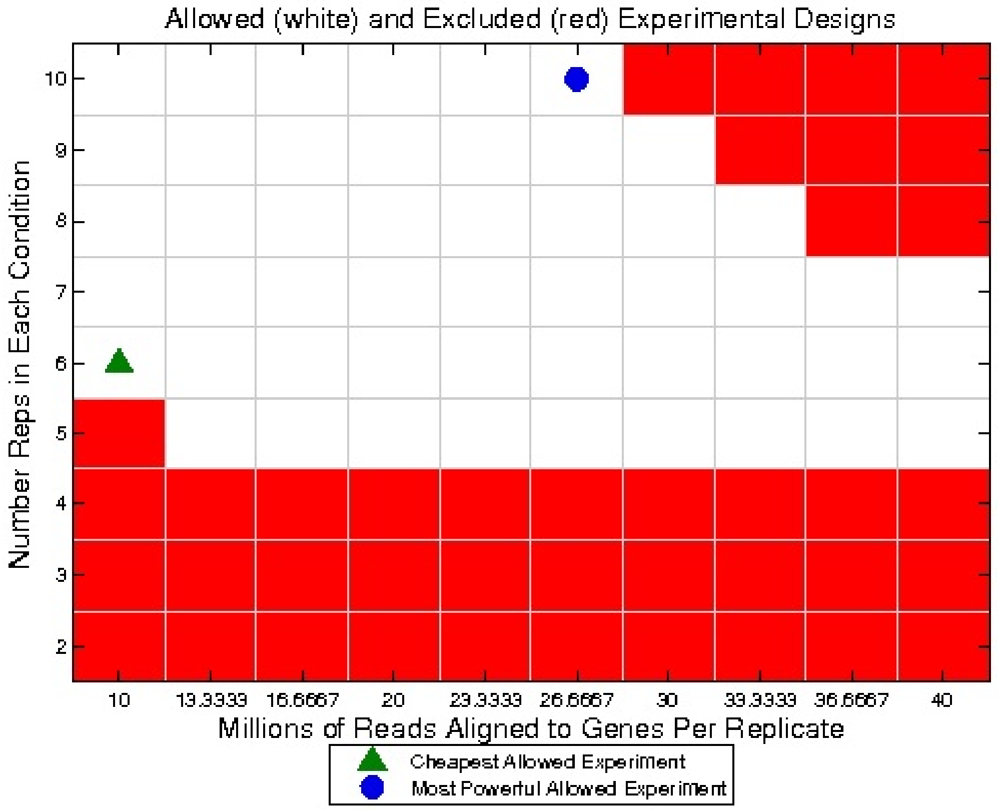

Predicted Statistical Power

[See example.](#)

The least expensive experiment that meets your criteria will yield the following power:

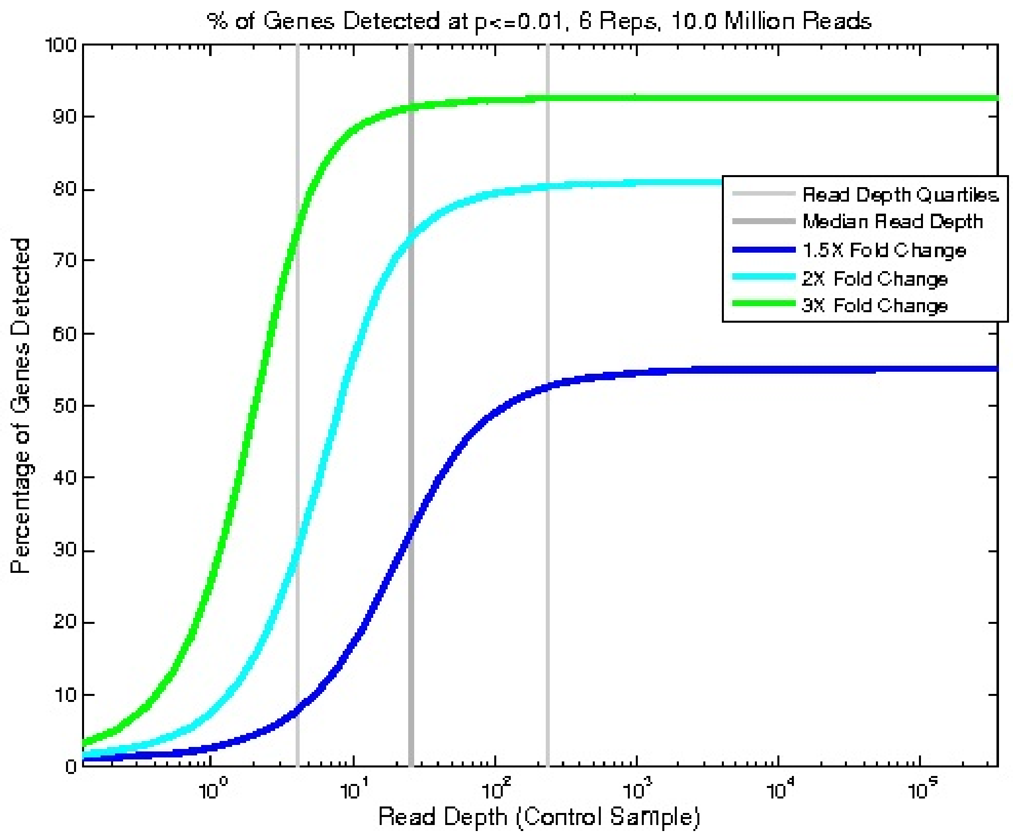

The most powerful experiment that meets your criteria will yield the following power:

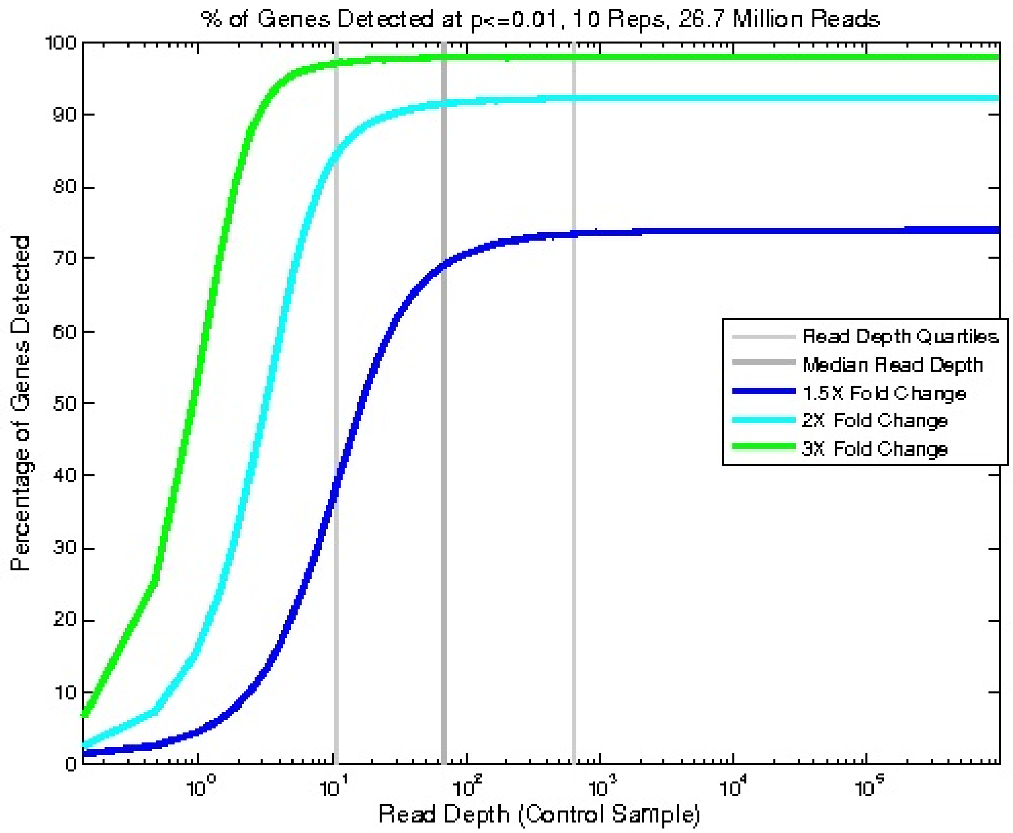

Thank you for using Scotty!
